# Burden of illness in Japanese patients with paroxysmal nocturnal hemoglobinuria receiving C5 inhibitors

**DOI:** 10.1007/s12185-023-03698-5

**Published:** 2024-01-19

**Authors:** Naoshi Obara, Kensuke Usuki, Takeshi Hayashi, Masato Fujii, Takayuki Ikezoe

**Affiliations:** 1 Department of Hematology, Faculty of Medicine, University of Tsukuba, Tsukuba, Japan; 2 Department of Hematology, NTT Medical Center Tokyo, Tokyo, Japan; 3 Sobi Japan, Tokyo, Japan; 4 Department of Hematology, Faculty of Medicine, Fukushima Medical University, Hikarigaoka‑1, Fukushima, Fukushima 960‑1295 Japan

**Keywords:** Paroxysmal nocturnal hemoglobinuria, Fatigue, Quality of life, Hemoglobin, Burden of illness

## Abstract

Paroxysmal nocturnal hemoglobinuria (PNH) is a rare, acquired, life-threatening blood disorder characterized by hemolysis and resulting in anemia and fatigue. Current therapies for PNH in Japan rely on complement inhibitors targeting the C5 component of the complement. However, the disease burden of Japanese patients with PNH treated with C5 inhibitors (C5i) remains unclear. To investigate this topic, we conducted a cross-sectional survey study that included 59 Japanese patients with PNH treated with C5i. Although many participants received C5i for 1 year or longer, the mean hemoglobin (Hb) level was 10.2 g/dL. Fatigue and shortness of breath were the most common symptoms at the time of diagnosis and survey. In addition, patients with Hb levels ≥ 10.5 g/dL also reported fatigue, depression and reduced quality of life, albeit to a lesser extent. These results suggest that a substantial burden of illness remains in patients with C5i-treated PNH, likely resulting in low quality of life and effects of symptoms on daily life. This study contributes to understanding the unmet needs of the current therapies for PNH, highlighting the need for novel therapeutics.

## Introduction

Paroxysmal nocturnal hemoglobinuria (PNH) is a rare, acquired, life-threatening blood disorder, affecting 12–13 per million people in the United States (US) [1], with a median age at diagnosis of around 30–50 years globally [2]. PNH is characterized by anemia, thrombosis, fatigue, dyspnea, abdominal pain, smooth muscle dystonia, renal failure, arterial and pulmonary hypertension, and recurrent infection [3, 4]. In PNH, hemolysis, which is the complement-mediated destruction of red blood cells (RBC), is induced by somatic mutations in the *PIGA* gene. In general, untreated patients with PNH mainly suffer from intravascular hemolysis (IVH) [5].

In Japan, PNH is treated with terminal complement inhibitors, which target the C5 component of the complement cascade, suppressing IVH [6]. Both eculizumab and ravulizumab are terminal complement inhibitors that can improve renal function and lower the thrombotic risk and mortality rate in patients with PNH [3, 7]. However, these inhibitors lead to the accumulation of C3b on the cell membrane, resulting in manifestation of extravascular hemolysis (EVH) in the spleen and other reticuloendothelial systems [8].

The diagnostic criteria for PNH are similar in Japan [2], the US, and Europe. Although no differences have been identified in the gender ratio, ethnic distribution, or geographic trends among these countries [9], there are variations in symptoms and age at diagnosis. A previous study suggested that symptoms of hematopoietic failure are predominant in Asians, including Japanese, whereas more classic PNH symptoms are observed in Western cases [2]. The median age at PNH diagnosis (initial visit) is 45 years in Japan and this is significantly older than the US median of 30 years, according to the Study Group on Idiopathic Hematopoietic Disorders [10]. This may be due to that the diagnosis is more likely to be delayed in Asian cases, especially in Japanese cases, because of the less pronounced nature of PNH symptoms such as thrombosis [2]. Actually, one study demonstrated that Japanese patients are less likely to develop thrombosis (6.2 vs. 19.3%, *p* < 0.0001) and have a longer mean survival time (32.1 vs. 19.4 years) compared to Caucasian patients [10]. Despite the differences in PNH characteristics, treatment with eculizumab or ravulizumab is indicated in the treatment guidelines used by all of these countries [2].

A patient-reported outcome (PRO) study from the US showed that patients with PNH treated with C5 inhibitors experience substantial burden of illness, indicating a need for improved treatment [11]. In this study, 87.5% and 82.9%, respectively, of eculizumab- and ravulizumab-treated patients remained anemic and 52.2% and 22.6%, respectively, of the patients receiving eculizumab or ravulizumab for ≥ 12 months still required transfusions within the past year. It is also reported that 51/141 (36%) patients received at least one transfusion, with 23 (16%) requiring 3 or more transfusions, and that 30 (21%) patients were receiving a higher dose of eculizumab (1200 mg or more every 2 weeks) [12]. Moreover, the majority of the patients reported fatigue symptoms and the level of quality of life (QoL) of the patients was lower than that of the general population. A similar study was conducted in Europe, which reported that PNH symptoms persist despite C5 inhibitor therapy (eculizumab or ravulizumab) and patients with PNH experience greater fatigue and poorer health-related QoL compared to the European general population references [13]. However, few studies have examined PROs and the disease burden on patients with PNH treated with eculizumab or ravulizumab in Japan. In this study, we aimed to quantify the disease burden on patients with PNH currently receiving eculizumab or ravulizumab treatment in Japan and to understand the unmet needs of these patients.

## Materials and methods

### Study design

This was a cross-sectional observational study of patients with PNH conducted in Japan via a survey delivered online and by mail. The study protocol was registered at the University Hospital Medical Information Network (UMIN) in Japan (UMIN ID 000050211). Participants were recruited from a PNH patient advocacy group. As there were 90 advocacy group members, the target sample size was set at 60, allowing for an estimated response rate of 30–70%.

### Participants and recruitment procedures

Participants were recruited via the PNH Club patient advocacy group. The inclusion criteria were as follows: (1) age ≥ 18 years, (2) a self-reported diagnosis of PNH, (3) a self-declaration stating that he/she is currently receiving eculizumab or ravulizumab, and (4) provided informed consent to participate in the study. Patients in the advocacy group received the survey link by e-mail invitation or the questionnaire by post. The invitation included instructions on completing the questionnaire online or on paper.

### Management of data and survey responses

In both the online and mail surveys, eligibility screening was built into the survey. In the online survey, respondents who did not satisfy the inclusion criteria were screened out after their response to the relevant question. In the mail survey, they were screened out during data processing. Duplication of responses was checked before analyses based on the respondents’ personal information.

### Survey content

Survey participants were asked for basic demographic information and were then questioned on five major PROs, which corresponded to the primary endpoints. These were: (1) Self-reported hematological and clinical measures (including a diagnosis of ongoing anemia, hemoglobin (Hb) level, history of RBC transfusions, history of thrombotic events, and history of renal function impairment); (2) QoL was assessed using the EQ-5D-5L measure and the European Organization for the Research and Treatment of Cancer (EORTC) QLQ-C30; (3) Levels of fatigue and depression were assessed using the Functional Assessment of Chronic Illness Therapy-Fatigue (FACIT-Fatigue) scale and the Patient Health Questionnaire-8 (PHQ-8) depression scale; (4) Impact of PNH on work productivity was assessed using the Work Productivity and Activity Impairment questionnaire (WPAI); (5) Self-reported health resource utilization.

The evaluation tools used in this study were as follows: (1) EQ-5D-5L descriptive system questionnaire [14] and a visual analogue scale (EQ VAS), (2) EORTC QLQ-C30 version 3.0 [15], (3) FACIT-Fatigue [16], (4) PHQ-8 [17, 18], and (5) WPAI absenteeism, presenteeism, work productivity loss, and activity impairment subscales [19]. General population values for the FACIT-Fatigue and EORTC QLQ-C30 assessments were derived from the available literature for comparison [16, 20].

### Ethical considerations

All study materials; including the study protocol, questionnaires, and informed consent forms; were approved by the Ethics Review Board of the Kitamachi Clinic (No. BGQ09129). The study was conducted in compliance with the approved protocol and according to the Ethical Guidelines for Medical and Health Research Involving Human Subjects (March 10, 2022), issued by the Ministry of Education, Culture, Sports, Science and Technology, Ministry of Health, Labor and Welfare, and the Ministry of Economy, Trade, and Industry in Japan and the tenets of the 2013 revision of the Declaration of Helsinki.

Participants were clearly and fully informed of the purpose of the study, potential risks, and their rights and responsibilities before enrollment in the study. Informed consent was obtained from all participants before study commencement.

### Statistical analysis

Descriptive statistics were performed to characterize patient demographics and PROs. Continuous variables were summarized using means, medians, and standard deviations (SDs). The categorical variables were summarized using counts and proportions. Some variables had missing values due to missing or inappropriate responses to the survey questions.

For additional subgroup analyses, we categorized those participants who had reported their Hb levels into two groups by level (Hb < 10.5 g/dL vs. ≥ 10.5 g/dL). A subgroup cut-off between levels of 10.5 g/dL was used because it reflects the median of real-world PNH populations and clinical trial inclusion criteria [12, 21, 22]. Subgroup analyses by age (by median age of the entire sample, and by < 65 vs. ≥ 65 years old), history of thrombotic events, and history of renal function impairment were also performed. All statistical analyses were performed using BellCurve Hideyoshi Dplus v.1.10 (Social Survey Research Information Co., Ltd. Tokyo, Japan), and BellCurve for Excel (Social Survey Research Information Co., Ltd. Tokyo, Japan).

## Results

### Cohort demographics and clinical/treatment characteristics

**Fig. 1:**
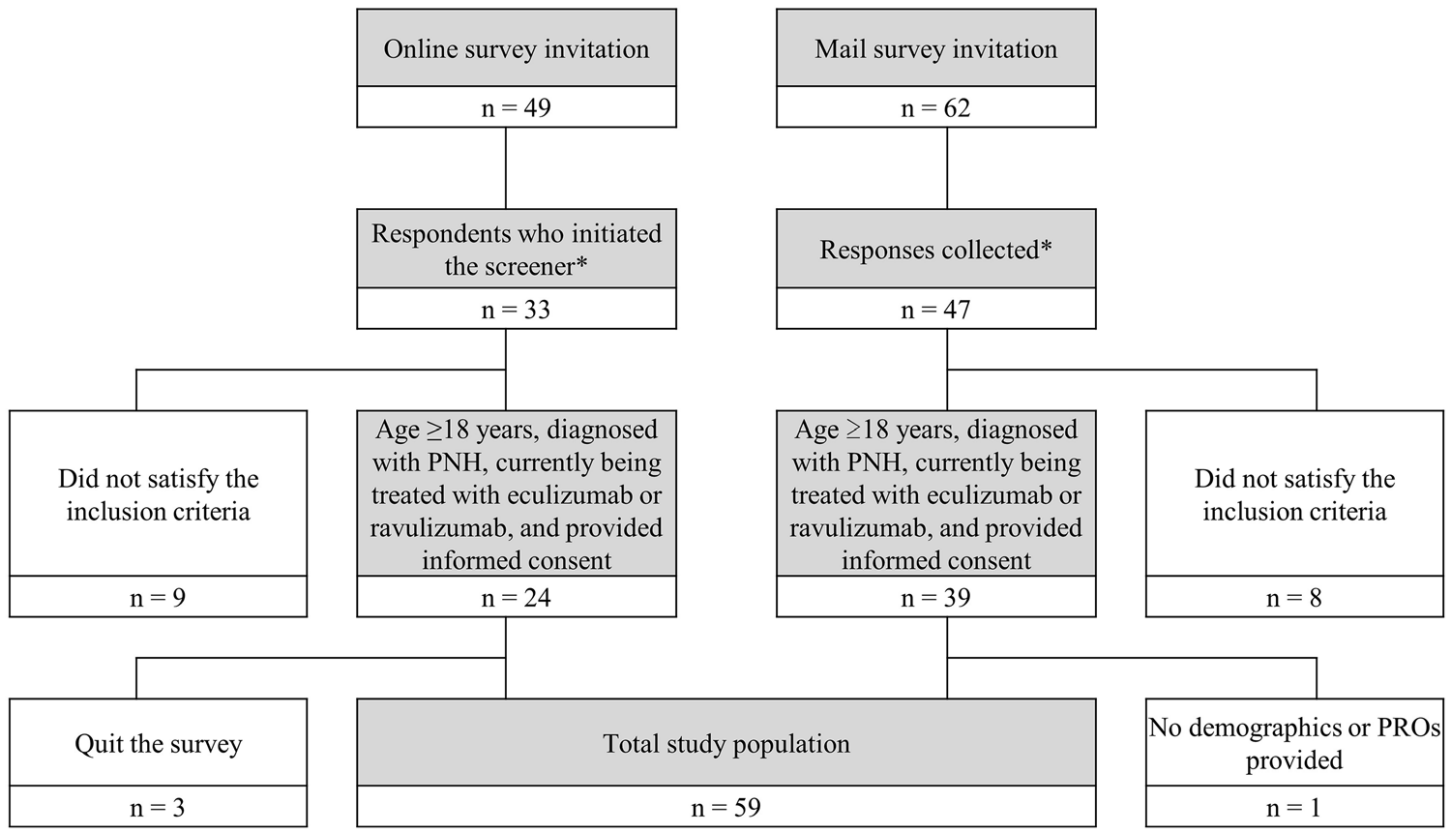
Patient selection for study participation. *No duplicate respondents were identified﻿

A total of 49 patients were invited by e-mail to participate in the survey. Of the 33 patients who responded and were screened by the online questionnaire, 24 satisfied the inclusion criteria. Three of these patients discontinued the survey before completion and were excluded from the analyses. A total of 62 paper-based questionnaires were sent directly to patients by post. Among the 47 responses collected, 39 satisfied the inclusion criteria. One of these was excluded because it did not provide demographic information or answers to the PRO questions. Ultimately, 59 participants (21 online and 38 postal) were included in the analyses (Fig. 1). No duplication was found in the collected responses by online and by post.

**Fig. 2:**
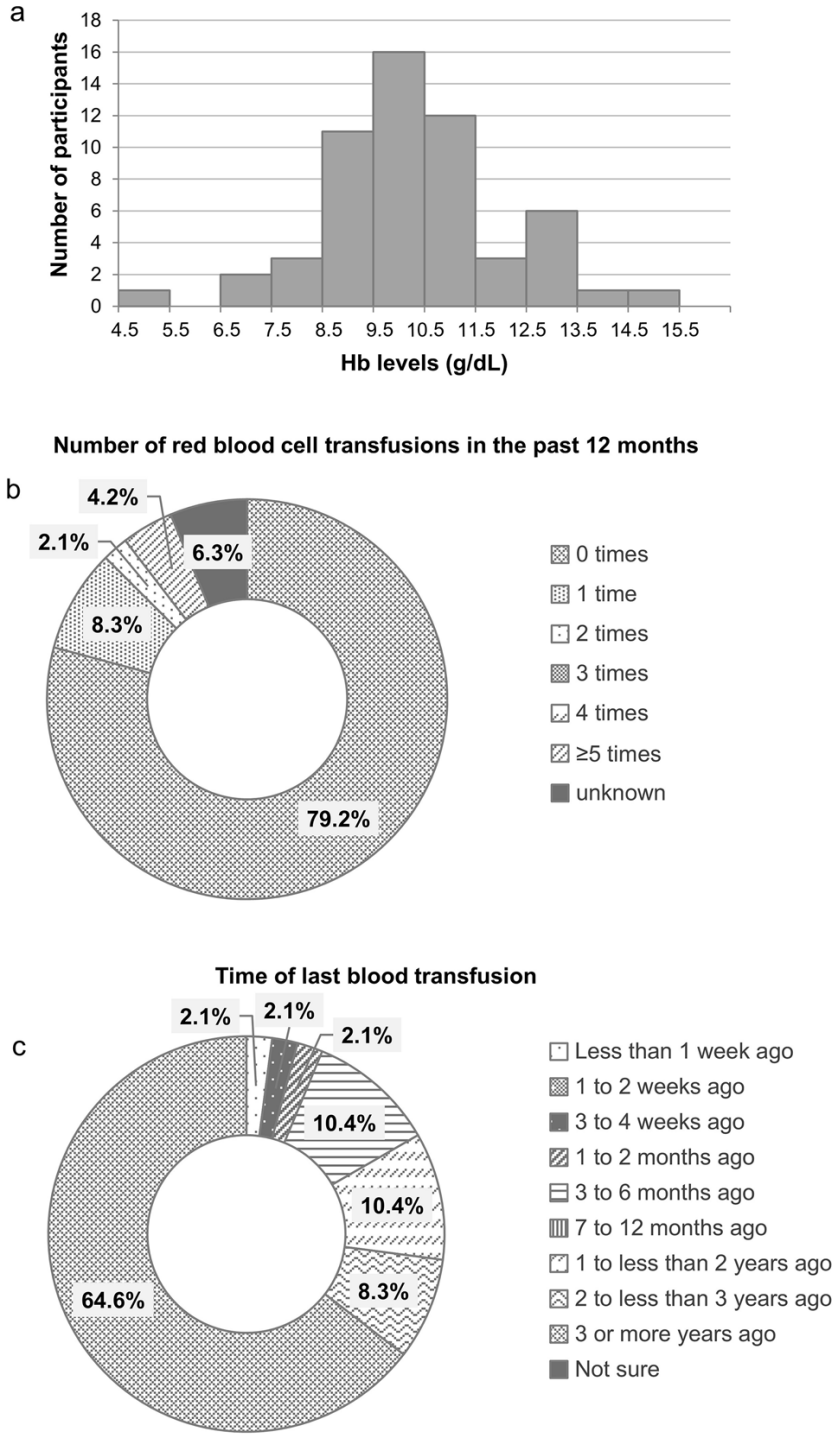
Hemoglobin levels and blood transfusion histories of patients with paroxysmal nocturnal hemoglobinuria receiving C5 inhibitor treatment. **a** Distribution of survey participant hemoglobin levels (without missing values, *n* = 56). **b** Number of red blood cell transfusions in the past 12 months (had ever experienced a blood transfusion, *n* = 48). **c** Time of last blood transfusion (had ever experienced a blood transfusion, *n* = 48). *Hb* hemoglobin

The demographic and clinical characteristics of the survey participants are presented in Table 1. The mean age (± SD) was 56.7 (± 14.3) years and 64.4% of the participants were female. The average weight (± SD) was 57.3 (± 10.2) kg. The average (± SD) duration between PNH diagnosis and survey participation was 16.8 (± 11.8) years (median: 13.0, interquartile range: 9.0–21.8). Most (*n* = 55) of the participants were receiving ravulizumab treatment, with just four on eculizumab. Most (*n* = 51) had been on C5 inhibitor therapy for at least a year. The mean (± SD) Hb level was 10.2 (± 1.8) g/dL. The distribution of Hb levels is presented in Fig. 2a. Of the 56 participants with available Hb data, 58.9% (*n* = 33) were classified into the lower Hb group (Hb < 10.5 g/dL). History of aplastic anemia or severe aplastic anemia was present in 39% of the participants. Although changes before and after C5 inhibitor therapy are not known from the survey, of the 81.4% (*n* = 48) who had undergone blood transfusions, 14.6% had undergone at least one transfusion in the past 12 months (Fig. 2b), whereas 64.6% had not undergone a blood transfusion in the past 3 years (Fig. 2c), regardless of the time of initiation of C5 inhibitor therapy. When stratified by Hb levels, at least one blood transfusion in the past 12 months was reported by 17.2% of the lower (< 10.5 g/dL) Hb group and 11.8% of the higher (≥ 10.5 g/dL) Hb group. The proportion of the participants who had not experienced blood transfusions in the past 3 years was 48.3% in the lower Hb group and 88.2% in the higher Hb group. Thrombotic events were reported by 15.3%, and a history of renal insufficiency was reported by 13.6% of the participants.

**Table 1:** Demographic and clinical characteristics of the survey participants

Characteristic	Mean (SD)	*n* (%)
Demographics		
Age (years)	56.7 (14.3)	−
Sex		
Male	−	20 (33.9)
Female	−	38 (64.4)
Unknown	−	1 (1.7)
Clinical characteristics		
Weight (kg)^a^	57.3 (10.2)	−
Age at diagnosis (years)^a^	39.4 (16.3)	−
Time from diagnosis (years)^a^	16.8 (11.8)	−
Time from C5i treatment initiation (months)		
0–2	−	3 (5.1)
3–11	−	4 (6.8)
12–23	−	13 (22.0)
24–35	−	20 (33.9)
≥ 36	−	18 (30.5)
Unknown	−	1 (1.7)
Comorbidities		
Aplastic anemia/severe aplastic anemia	−	23 (39.0)
Myelodysplastic syndrome	−	5 (8.5)
Other bone marrow disorder	−	1 (1.7)
None of the above	−	26 (44.1)
Unknown	−	5 (8.5)
Most recent hemoglobin level (g/dL)^b^	10.2 (1.8)	−
Had ever been diagnosed with anemia (without diagnosis of aplastic anemia; *n* = 31)	−	25 (80.6)
Had ever experienced a blood transfusion	−	48 (81.4)
Had ever experienced a thrombotic event	−	9 (15.3)
Have a history of renal insufficiency	−	8 (13.6)
Current treatment		
Ravulizumab	−	55 (93.2)
Eculizumab	−	4 (6.8)

*C5i* C5 inhibitor

^a^One participant was excluded because of a missing value

^b^Three participants were excluded because of a missing value

### Quality of life

**Fig. 3:**
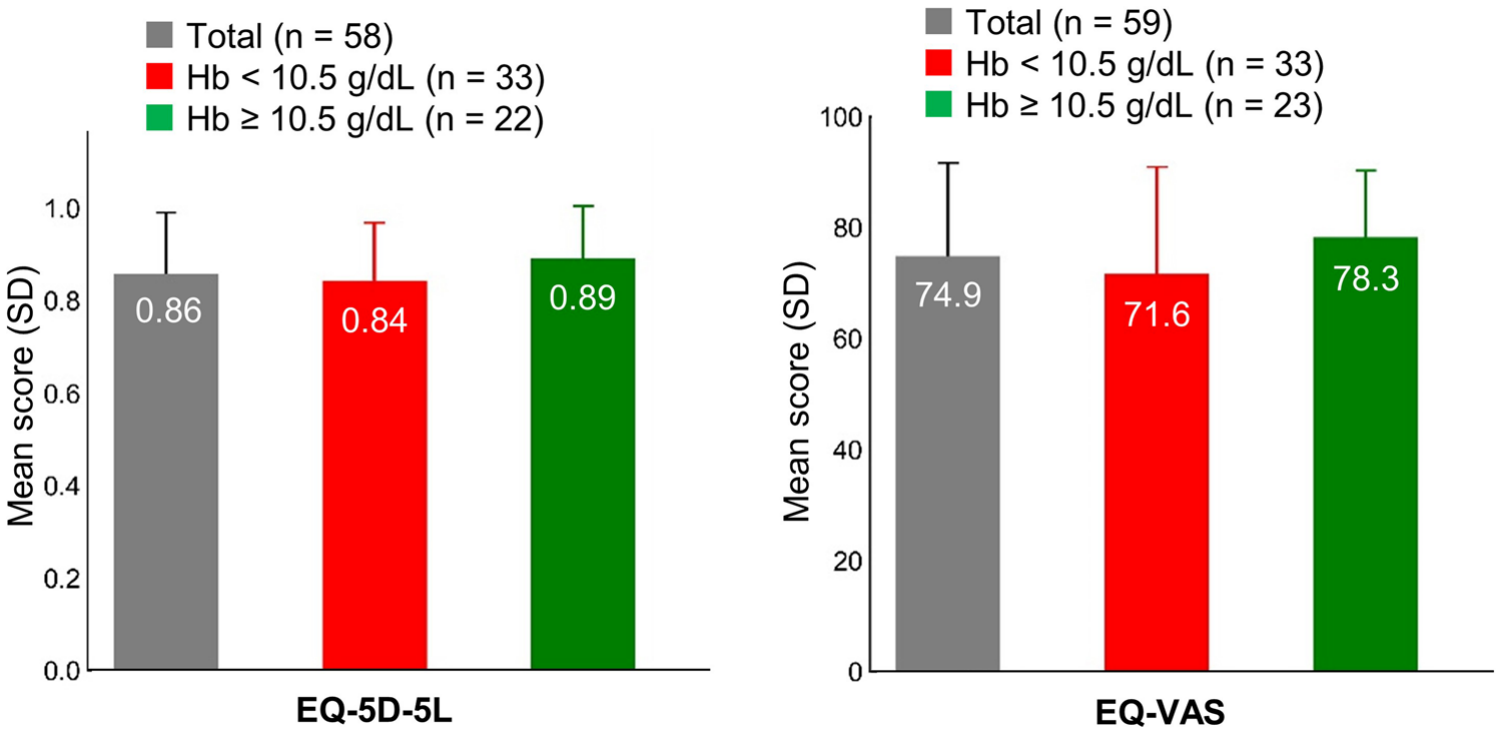
EQ-5D-5L index and EQ VAS scores of patients with paroxysmal nocturnal hemoglobinuria receiving C5 inhibitor treatment. *Hb* hemoglobin, *SD* standard deviation

The mean (± SD) EQ-5D-5L index value of the cohort was 0.86 (± 0.13) and the mean (± SD) EQ VAS score was 74.9 (± 16.8). The mean (± SD) EQ-5D-5L index values in the lower and higher Hb subgroups were 0.84 (± 0.13) and 0.89 (± 0.11), respectively. The mean (± SD) EQ VAS scores of the lower and higher Hb subgroups were 71.6 (± 19.3) and 78.3 (± 12.0), respectively (Fig. 3). The EQ-5D-5L index values generally range from < 0 (where 0 is the value of a health state equivalent to dead, and negative values represent values that are considered worse than dead) to 1 (full health), with higher scores indicating higher health utility [23]. On EQ VAS, the participants can rate their perceived health from 0 (worst imaginable health) to 100 (best imaginable health).

**Fig. 4:**
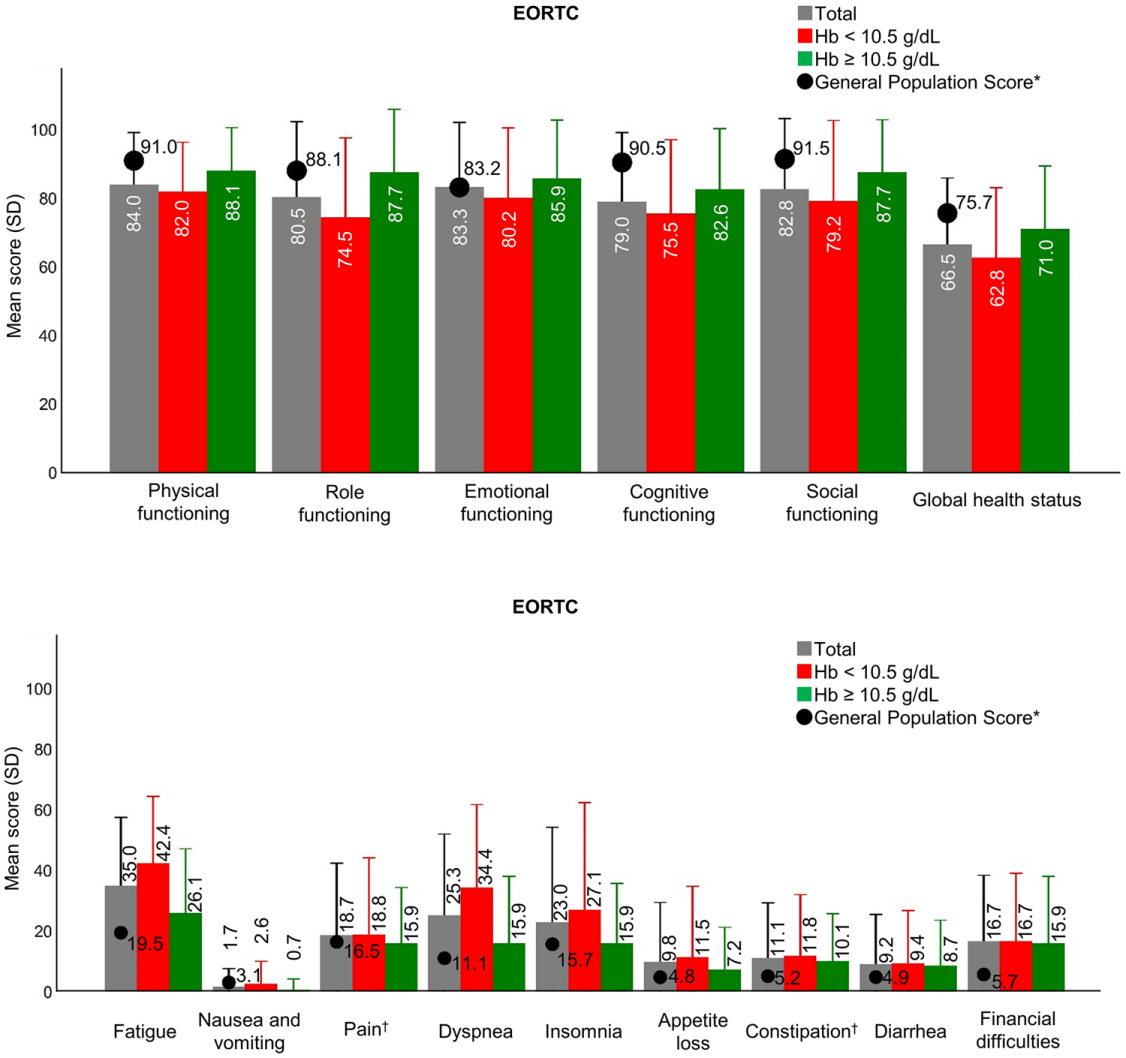
European Organization for Research and Treatment of Cancer QLQ-C30 scores (means and standard deviations) of patients with paroxysmal nocturnal hemoglobinuria receiving C5 inhibitor treatment. ^*^General population scores were derived from Hinz et al. [20]. Unless otherwise noted, sample sizes were *n* = 58 for the total study population, *n* = 32 for the Hb < 10.5 g/dL subgroup, and *n* = 23 for the Hb ≥ 10.5 g/dL subgroup. ^†^*n* = 57 for total study population, *n* = 31 for Hb < 10.5 g/dL, and *n* = 23 for Hb ≥ 10.5 g/dL. *Hb* hemoglobin, *SD* standard deviation

The mean (± SD) global health status scores of the entire cohort on the EORTC QLQ-C30 was 66.5 (± 19.5), compared to a mean general population score of 75.7 [20]. Participants with lower Hb levels showed lower scores on the functional scales, and higher scores on the symptom scales (Fig. 4). All EORTC QLQ-C30 scale scores range from 0 to 100 points. A high score for a functional scale represents a high/healthy level of functioning, a high score for the global health status/QoL represents a high QoL, but a high score for a symptom scale/item represents a high level of symptomatology/problems [24].

### PNH symptoms and depression

**Fig. 5:**
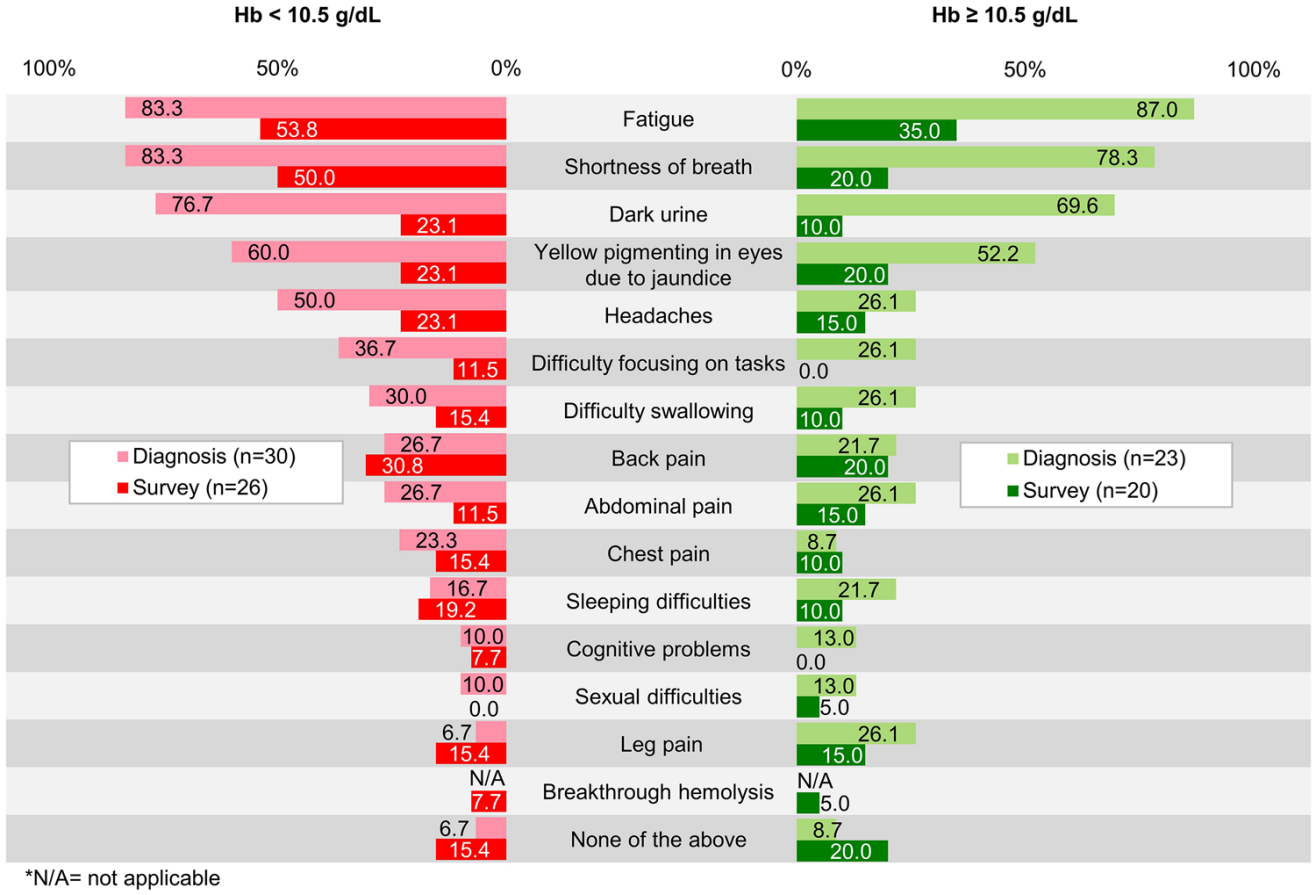
Symptoms at diagnosis and the time of the survey of patients with paroxysmal nocturnal hemoglobinuria receiving C5 inhibitor treatment. *Hb* ﻿hemoglobin

Figure 5 shows the frequently reported symptoms in the two Hb subgroups. At the time of diagnosis (*n* = 53), common symptoms were fatigue (*n* = 45, 84.9%), shortness of breath (*n* = 43, 81.1%), and dark urine (*n* = 39, 73.6%). At the time of the survey (*n* = 46), fatigue (*n* = 21, 45.7%) and shortness of breath (*n* = 17, 37.0%) remained the most common symptoms overall.

**Fig. 6:**
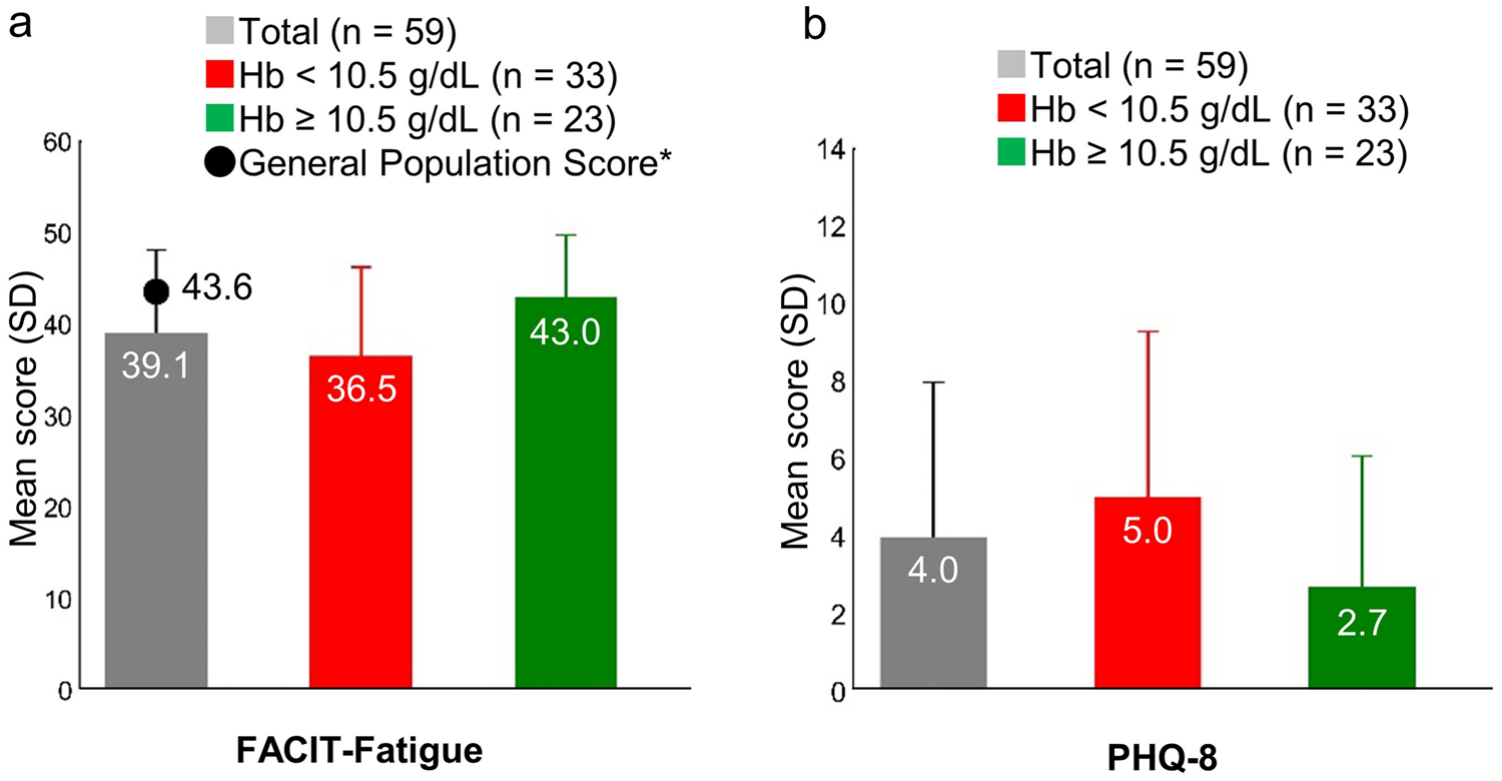
Functional assessment of chronic illness therapy-fatigue and Patient Health Questionnaire-8 scores of patients with paroxysmal nocturnal hemoglobinuria receiving C5 inhibitor treatment. ^*^General population score was derived from Cella et al. [16]. Total study population includes three participants with missing Hb levels. *Hb* hemoglobin, *SD* standard deviation

The average (± SD) score of the survey participants on the FACIT-Fatigue scale was 39.1 (± 9.1). The FACIT-Fatigue scale score ranges from 0 (worst) to 52 (no fatigue), with 52 as the best possible score [25]. Those with higher Hb levels had a mean (± SD) score of 43.0 (± 6.8), which is similar to that of the general US population (43.6 ± 9.4) [16]. On the contrary, those with lower Hb levels had a mean (± SD) score of 36.5 (± 9.7) (Fig. 6a).

The mean (± SD) PHQ-8 depression score of the cohort was 4.0 (± 4.0). The range of PHQ-8 total score is between 0 and 24 points. A total score of 0 to 4 represents no significant depressive symptoms; 5 to 9, mild; 10 to 14, moderate; 15 to 19, moderately severe; and 20 to 24, severe [17]. The mean (± SD) scores of the subgroups with lower and higher Hb levels were 5.0 (± 4.3) and 2.7 (± 3.4), respectively (Fig. 6b).

### Impact of PNH on work productivity

**Fig. 7:**
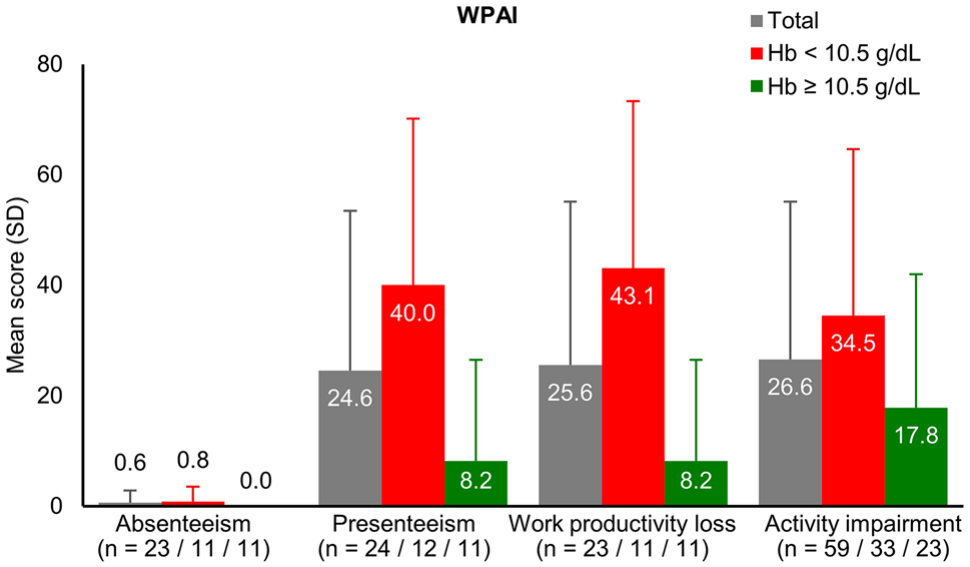
Work productivity and activity impairment among patients with paroxysmal nocturnal hemoglobinuria receiving C5 inhibitor treatment. *Hb* hemoglobin, *SD* standard deviation

Of the 59 survey participants, 25 (42.4%) were working for pay at the time of the survey. For those working, an average of 0.6% of working hours were missed due to PNH-related problems. The average level of presenteeism (affected productivity) was 24.6% of working hours. Among the total study population (with or without work pay), the mean level of activity impairment was 26.6% (Fig. 7).

### Health resource utilization

At least one emergency room (ER) visit in the last 12 months due to PNH-related problems was reported by 5.1% (*n* = 3) of the participants. PNH-related hospitalizations in the last 12 months were reported by 13.6% (*n* = 8) of the participants.

## Discussion

This study investigated the burden of illness in patients with PNH treated with C5 inhibitors in Japan. The participants were diagnosed with PNH for an average of 16.8 years before the study, and > 80% had been on anti-C5 therapy for > 1 year (Table 1). However, despite treatment, the mean Hb level of the cohort was 10.2 g/dL, and 33 participants (58.9%) reported Hb levels < 10.5 g/dL (Fig. 2a). The results indicated that a substantial disease burden remains in patients with PNH receiving anti-C5 treatment, not only in patients with Hb levels below 10.5 g/dL but also in patients with Hb levels ≥ 10.5 g/dL, which resulted in low QoL and a great symptomatic impact on daily life.

A US study on all-cause healthcare resource utilization found an average of one ER visit and one hospitalization related to PNH in the last 12 months [11]. However, the majority of the participants in our study had not utilized health resources for their PNH in the last 12 months, including ER visits and hospitalizations.

The mean QoL of our patients with PNH in Japan, as measured with the EQ-5D-5L and EORTC, was lower than that of the general population. A previous study reported that EQ-5D-5L scores of Japanese population aged 50–59 years were 0.936 for men and 0.928 for women [26]. This was a comparable age group as the mean age of the participants in our study was 56.7 years. The mean EQ-5D-5L score of our cohort was 0.86, which was lower than that of the general population. The mean score on the global health status scale of the EORTC QLQ-C30 in this study was 66.5, which was similar to the scores of patients with PNH reported in previous studies in Europe (66.9) and the US (62.4 in the eculizumab group and 67.2 in the ravulizumab group) [11, 13]. Although there are some differences in clinical characteristics between Japanese and Caucasian patients, such as thrombosis and mean survival time, it is reported that interpretation of EORTC QLQ-C30 is not affected by Asian ethnicity [27]. Our results showed that Japanese patients with PNH had a similar level of QoL compared to that of PNH-patients in Europe or the US and that irrespective of the severity of the anemia PNH-patients treated with C5 inhibitors had reduced QoL compared with the general population.

The responses of our cohort on the EORTC QLQ-C30 subscales indicated that the levels of fatigue, dyspnea, and insomnia are particularly high in patients with PNH and likely contribute to their reduced QoL. Fatigue and shortness of breath were the most frequently occurring symptoms, which were reported at the time of diagnosis. These remained the most common symptoms at the time of the survey. Fatigue at the time of the survey was reported by 53.8% and 35.0% of the patients in the lower and higher Hb subgroups, while shortness of breath was reported by 50.0% and 20.0% in the two subgroups, respectively. All of the patients in our sample were receiving C5 inhibitor therapy, but the treatment did not sufficiently improve these symptoms and QoL.

More than half of the patients with low Hb levels at the time of the study, experienced fatigue. While the fatigue levels, as measured on the FACIT-Fatigue scale score, of the higher Hb subgroup were comparable (43.0) with those of the general population (43.6) [16]; the lower Hb group scored an average of 7.1 points less (36.5, lower scores indicate worse fatigue) than the general population, a difference greater than the clinically meaningful difference in a patient with PNH (a ≥ 5 points change in score is considered clinically meaningful) [28].

Previous studies have shown correlations between QoL and depression scores. For example, a study in Japan found that depression, measured using the Hospital Anxiety and Depression Scale, was significantly correlated with QoL, measured by EORTC QLQ-C30 functioning and global scores, in cancer patients [29]. In our study, the mean PHQ-8 score of patients with PNH was 4.0, and scores of 0–4 represent no significant depressive symptoms [17, 18]. However, the mean score of the lower Hb level subgroup was 5.0, and scores of 5–9 on this measure denote mild depressive symptoms. Within the lower Hb group, 15 patients (45.5%) had scores indicative of mild to moderately severe depressive symptoms.

As measured by the WPAI tool, the effect of PNH on work productivity was an absenteeism rate of 0.6% and a presenteeism rate of 24.6%. Compared with the rates reported in other countries, the absenteeism in this study was minimal. From our result, absenteeism due to PNH would appear to occur relatively rare in Japan. Our presenteeism rate was higher than that found in a European study (18.7%) [13] but lower than the rate in the US (31.5%) [11]. This result should be interpreted with caution as only 56.1% of those less than 65 years old in our cohort were in paid employment. This was 54.5% in the lower Hb subgroup and 58.8% in the higher Hb subgroup. Those patients most severely affected by PNH symptoms may not be in employment so the rates of presenteeism and absenteeism could lead to an underestimation of the impact of PNH on work productivity.

Activity impairment in the study participants, as measured by WPAI, was 26.6%, on average. This was lower than the rates reported in both Europe (39.7%) and the US (39.3%) [11, 13]. The level of impairment in performance of daily activities among patients with PNH, including those not in employment, was interpreted as low in Japan. However, the mean age ± SD of the participants in this study was approximately 10 years older than for participants in the European and US research (56.7 ± 14.3 years in this study; 43.0 ± 13.1 years in Europe; and 46.8 ± 15.7 years in the US) [11, 13]. Furthermore, the most frequently observed symptoms in the present study were fatigue and shortness of breath. Taken together, this could mean that the participants had misattributed PNH symptoms as age-related, which could have caused them to underestimate the degree of impairment due to PNH. Future studies with larger sample sizes are required, with subgroup comparisons between different age groups.

This study had some limitations. First, due to the cross-sectional design, we cannot ascribe cause-and-effect relationships, just associations, between variables. Second, because the participants of this study were recruited via a patient advocacy group rather than by random selection, volunteer bias may have occurred, potentially limiting the generalizability of the results. In particular, this may mean that the percentage of employed patients with PNH in our cohort does not reflect the percentage of working patients with PNH in Japan. However, demographics such as age and sex in our study population did not differ largely from the distributions in PNH-patients treated with anti-C5 in other research [30]. In addition, patients who belong to patient advocacy groups may be more likely to recognize the symptoms of PNH, and thus the results of this study may be biased toward such patients. Third, all study data were obtained from self-administered questionnaires, so the medical information could not be confirmed by clinicians. Therefore, the survey responses could be subject to recall bias and misclassification and the results regarding medical information such as Hb levels and history of RBC transfusions should be interpreted with caution. Also, special attention should be paid to the interpretation of history of RBC transfusion in past years because it includes the data for blood transfusion prior to receiving C5 inhibitor treatment. Moreover, clinical information other than a diagnosis of ongoing anemia, Hb level, history of RBC transfusions, history of thrombotic events, and history of renal function impairment could not be collected and evaluated. Fourth, the clinically significant cut-off of Hb levels among PNH-patients who have a suboptimal response to C5 inhibition is controversial. Some of the clinical trials with PNH-patients have used < 10 g/dL or ≤ 9.5 g/dL as the inclusion criteria [31, 32]. The 10.5 g/dL threshold used in our study has been discussed as a clinically validated cut-off by the National Institute for Health and Care Excellence in the United Kingdom [33]. However, its clinical significance remains unclear. Finally, due to the limited sample size in this study, our subgroup analyses, other than those for Hb levels, comprised groups too small for the results to be sufficiently valid and reliable. Therefore, the comparisons of other subgroups were not included in the results section.

This study has shown that many patients with PNH in Japan treated with eculizumab or ravulizumab still suffer a significant disease burden in terms of symptoms, QoL, work productivity, and health resource utilization. It is hoped that this research highlights the need of novel more effective therapeutics for the treatment of PNH.

## Data Availability

The datasets generated during and/or analyzed during the current study are available upon reasonable requests, from the corresponding author or by sending a data sharing request form (available on www.sobi.com) to medical.info@sobi.com. All requests are evaluated by a cross‐functional panel of experts within Sobi, and a decision on sharing will be based on the scientific merit and feasibility of the research proposal, maintenance of personal integrity, and commitment to publication of the results. Further information on Sobi’s data sharing policy and process for requesting access can be found at: https://www.sobi.com/en/policies.
